# Local tissue electrical parameters predict oral mucositis in HNSCC patients: A diagnostic accuracy double-blind, randomized controlled trial

**DOI:** 10.1038/s41598-020-66351-9

**Published:** 2020-06-12

**Authors:** Gabriela Luize Guimarães Sanches, Agna Soares da Silva Menezes, Laércio Ives Santos, Cristina Paixão Durães, Larissa Lopes Fonseca, Marcelo Perim Baldo, Thais de Oliveira Faria, Luciano Alves de Araújo Andrade, Petr Iakovlevitch Ekel, Sérgio Henrique Sousa Santos, Alfredo Maurício Batista de Paula, Lucyana Conceição Farias, Marcos Flávio Silveira Vasconcelos D’Angelo, André Luiz Sena Guimarães

**Affiliations:** 1 Department of Dentistry, Universidade Estadual de Montes Claros, Minas Gerais Montes Claros, Brazil; 2 Department of Computer Science, Universidade Estadual de Montes Claros, Minas Gerais Montes Claros, Brazil; 3 Department of Pathophysiology, Universidade Estadual de Montes Claros, Montes Claros, Minas Gerais Brazil; 4 Instituto Federal do Norte de Minas Gerais, Montes Claros, Minas Gerais Brazil; 5 Institute of Agricultural Sciences, Universidade Federal de Minas Gerais (UFMG), Montes Claros, Minas Gerais Brazil; 6 Dilson Godinho Hospital, Montes Claros, Minas Gerais Brazil; 7 Pontifícia Universidade Católica de Minas Gerais, Programa de Pós-Graduação em Engenharia Elétrica, Belo Horizonte, Minas Gerais Brazil

**Keywords:** Cancer, Medical research, Physics

## Abstract

Oral Mucositis (OM) is a common adverse effect of head and neck squamous cell carcinoma (HNSCC) treatment. The purpose of this study was to investigate the significance of early changes in tissue electrical parameters (TEPs) in predicting the development of OM in HNSCC patients receiving radiation therapy (RT). The current study combined two study designs. The first was a case-control study. The control group comprised of RT patients who did not receive head and neck RT, and patients with HNSCC who received RT comprised the case group. In the second part of the study, the case group was included in a parallel cohort. A total of 320 patients were assessed for eligibility, and 135 patients were enrolled. Double blinding was performed, and neither the patients nor the care providers knew the measured parameters. The primary outcome was the detection of between-group changes in local TEPs over the follow-up period. The secondary outcome was the appearance of OM grades II, III, or IV and the predictive value of local TEPs in determining the incidence of OM after RT. The variables, impedance module, resistance, reactance, phase angle, and capacitance, were analyzed by the receiver operator curves (ROC). The case and control groups did not differ in demographic and clinical characteristics. Radiation therapy increased the local impedance module, resistance, reactance, and phase angle and reduced the local tissue capacitance in both groups. Evaluation of TEPs in the first week of RT correlated with the development of OM lesions during cancer therapy. ROC analysis showed that local impedance module and resistance presented higher specificity than did other parameters in predicting OM. In conclusion, local tissue electrical parameters measured at the first RT week can be useful tools to predict oral mucositis.

## Introduction

Therapeutic approaches for head and neck squamous cell carcinoma (HNSCC) include surgery, radiation therapy (RT), chemotherapy alone, or in combination, based on clinical staging^1,2^, being associated with relevant sequelae of treatment^3^, which negatively impact patients’ lifestyle and well-being^4–6^. Moreover, HNSCC treatment is generally associated with high costs for patients and public health services^7^.

Treatment of HNSCC has some side effects that are painful and difficult to manage^8,9^. Oral mucositis (OM), dry mouth, sticky saliva, difficulty in swallowing solid foods, and loss of taste sensation are frequent complications of HNSCC RT treatment^10^. Oral mucositis is classified into 4 grades according to severity^11,12^. Grade 2 lesions are associated with pain, but the patient can still swallow solids. In grades 3 and 4, the discomfort interferes with food intake^10^. Oral mucositis may necessitate modifications in the RT fraction regimen as high grades of OM need treatment interruption^13^. Discontinuation of RT is associated with a decrease in local control rates and reduced survival^14,15^. Despite the impact of OM in the patient’s quality of life, there is no test to predict the incidence of OM during cancer therapy.

Many physiological processes are associated with electrical activity^16^, and some of these electrical parameters have proved to be useful in differentiating between normal and pathological processes^17–21^. TEPs are different and useful identifying tissues such as adipose and muscular tissue^17–20^. The majority of the studies have attempted to investigate only impedance as a marker of biological processes^17–23^. For example, impedance module and phase angle are widely used to evaluate body adiposity^24^ or, in a few cases, to detect neoplasms^21^.

It is known that RT alters the tissue concentrations of free radicals^25^, thus changing TEPs during RT^26^. However, no study has attempted to evaluate the local changes in impedance module, resistance, reactance, phase angle, and capacitance caused by RT. Moreover, there is no information on the association between TEPs and OM. Thus, the current study aimed to evaluate the significance of early changes in TEPs in predicting the development of OM in HNSCC patients receiving RT.

## Patients and methods

### Ethical approval

All performed procedures were conducted following the ethical standards of the institutional and national research committees, the 1964 Helsinki declaration, and its later amendments, or comparable ethical standards. Ethical approval for this study (Number 80731617.0.0000.5146) was obtained from the Universidade Estadual de Montes Claros Institutional Review Board. The study was also registered in the National Clinical Trials Network (UTN: U1111-1214-7398/RBR-7yygb2). Data were collected at Dilson Godinho Hospital in Brazil from March 2017 to July 2018. The trial protocol can be assessed at http://www.ensaiosclinicos.gov.br/, and ethical approval can be evaluated at http://plataformabrasil.saude.gov.br/. All patients signed the informed consent forms.

### Study design

The current study followed a double-blind, prospective, diagnostic, randomized controlled cohort design^27^. The study was reported following the Standards for Reporting Diagnostic Accuracy (STARD) criteria^28^ (Supplementary Material 1).

### Sample size calculation, allocation concealment, and blinding

The sample calculation was based on previous studies^29,30^ and hospital statistics. The sample size calculation was performed to achieve an alpha of 0.05, a beta of 0.05, and a study power of 0.95, and reach a minimal between-group difference of 50% in OM incidence. A total of 320 patients were assessed for eligibility, and 135 patients were enrolled in the study.

The study recruiter and the researcher involved with statistical analysis were not care providers. The random allocation sequence was performed not for the study recruiter. The allocation ratio was 1:1.25. Double-blinding was achieved, and neither the patients nor the care providers knew the measured parameters. The data were collected between March 2017 and July 2018 at Hospital Dilson de Quadros Godinho, located at Montes Claros city, state of Minas Gerais, Brazil.

### Groups

The case group comprised of patients with HNSCC. The inclusion criteria for the case group were adults older than 18 years (both sexes) with confirmed histopathological diagnosis of squamous cell carcinoma of the base of the tongue, malignant neoplasms of other or unspecified parts of the tongue, unspecified, squamous cell carcinoma of the gum, and squamous cell carcinoma of the floor of the mouth, the palate, other or unspecified parts of the mouth, the tonsil, the oropharynx, the piriform sinus, the hypopharynx, and its variants, other or ill-defined sites in the lip, oral cavity, or pharynx receiving RT alone or in combination with chemotherapy. All patients treated with RT received 3D-RT. Patients were excluded if they had previously received head and neck RT, presented with OM, received pacemakers or declined enrollment.

The control group included patients who received RT for prostate, urinary bladder, rectum anus, breast, and cervical cancer. The inclusion criteria for the control group were adults over 18 years of age (both sexes) with confirmed histopathological diagnosis of malignant neoplasms of the prostate, adenocarcinoma of the prostate, metastasis in other or unspecified organs of the urinary system, prostate carcinoma *in situ*, malignant neoplasms of the penis, unspecified, squamous cell carcinoma *in situ* of the mucocutaneous epithelium of the penis, other specified malignant neoplasms of the anus and anal canal, squamous cell carcinoma of the cervix uteri, adenocarcinoma of the cervix uteri, malignant neoplasms of the esophagus, unspecified, ductal carcinoma *in situ* of the breast, other specified malignant neoplasms of the breast and unspecified carcinoma of unspecified sites receiving RT alone or in combination with chemotherapy. All patients treated with RT received 3D-RT. Patients were excluded if they had previously received head and neck RT, presented with OM, received pacemakers or declined enrollment.

### Follow-up

The case group was followed during the RT treatment (approximately 60 days). Both primary and secondary outcomes were evaluated in the case group weekly right after the start of RT treatment until the end of RT. In the case of OM occurrence, treatment was initiated following standard protocols as described elsewhere^11,12^.

### Outcomes

The primary outcome was a change in local TEPs (impedance module, resistance, reactance, phase angle, and capacitance). The examination was done weekly right after the start of RT treatment until the end of RT.

The secondary outcome was the incidence of OM grades II, III, or IV, identified as previously described^11,12^. The presence of pain and the clinical appearance of mucositis lesions were detected during clinical examination, which was also performed weekly right after the start of RT treatment until the end of RT.

### Electrical parameters

The electrical resistance properties of the tissues were evaluated based on the following variables: impedance module, resistance, reactance, phase angle, and capacitance^20,23,24^. Impedance (Z) is an expression of the opposition that a system offers to alternating electric current^31^. Resistance (R) is a measure of the extent to which a substance opposes the movement of electrons among its atoms^31^. Reactance (X) is the amount of energy that a circuit stores^31^. When an alternating current passes through a body that contains reactance, the energy is released in the form of an electric field, in which case the reactance is capacitive (denoted -jX_C)^31^. Reactance is conventionally multiplied by the positive square root of −1, which is the imaginary unit number called the j operator, to express Z as a complex number of the form R- jX_C (when the reactance is capacitive)^31^. The impedance Z can also be represented in the form of impedance module (|Z | ) and phase angle (θ), where |Z | = √(R^2 + X^2) and θ = tan^(−1) (X/R)^31^. The capacitive reactance is given by X_C = (1/(2πfC)), where f is the frequency of the alternating current, and C is the capacitance. Capacitance is the ratio of the change in the electric charge of a system to the corresponding change in its electrical potential^31^. The concepts are summarized in Table 1.

**Table 1:** Relevant concepts.

Relevant concepts
Impedance (Z) is an expression of the opposition that a system offers to alternating electric current.
Resistance (R) is a measure of the extent to which a substance opposes the movement of electrons among its atoms.
Reactance (X) is the amount of energy that a circuit stores^31^.
Capacitance (C) is the ratio of the change in the electric charge of a system to the corresponding change in its electric potential.

Local TEPs (impedance module, resistance, reactance, phase angle, and capacitance) were measured in the irradiated area. Differently, systemic TEPs were collected from the whole body. Local and systemic TEPs were collected from case and control groups.

Systemic tetrapolar bioimpedance was evaluated as described elsewhere^20,23,24^. Briefly, a calibrated Bioelectrical Impedance Analysis Analyzer Unit (RJL Systems, Quantum BIA 101Q, Clinton Township, Michigan, EUA) using an electric current (400 mA) at a high frequency (50 kHz), between 0 and 1 ohms, was used to measure bioimpedance. Electrodes (Electrodes MSGST-06, Medico Electrodes International, Uttar Pradesh, India) were placed on the right hand, wrist, foot, and ankle (Supplementary Material 2). The local tetrapolar bioimpedance was evaluated bilaterally using the ala of the nose and the tragus as reference points (Supplementary Material 2). Both local and systemic TEPs were assessed in an environment at an appropriate temperature on clean, healthy skin. The patient was held in a supine position on a nonconductive surface, arms separated from the trunk at an angle of 30° and legs at 45°. Patients were instructed to avoid exercise, saunas, alcohol, and caffeine 8 hours before the procedure. They were advised not to eat or drink anything for one hour before the procedure, empty the bladder before the examination, and remove all metallic objects such as jewelry, rings, bracelets, and watches^20,23,24^. In both systemic and local bioimpedance, the wires generating the electric current were attached to the distal electrodes. Also, cables that detected voltage drop were added in the proximal. The vectors of resistance (R) and reactance (Xc), were used to derive mathematical equations to determine the phase angle (θ = tan^(−1) (Xc/R)), impedance module (Z = √(R^2 + X^2)), and capacitance (Xc = 1/((2π *f *C))).

### Oral mucositis grading

Patients were evaluated weekly, and mucositis was graded according to the World Health Organization (WHO)^12,32^. The same dentist performed all OM gradation.

### Statistical analyses

Data are shown as the mean ± standard deviation for continuous variables and as frequency and proportion for categorical variables. To determine if the data were well-modeled by a normal distribution, the Kolmogorov-Smirnov test and the Shapiro-Wilk test were performed. These analyses revealed that the data were non-parametrically distributed. Therefore, the Mann-Whitney test was performed. Pearson correlation was performed to evaluate the changes in local and systemic tissue electric parameters.

The variables, impedance module, resistance, reactance, phase angle, and capacitance, were tested by plotting the empirical Receiver Operating Characteristic (ROC) curves using the gold standard method for comparison. The definition of areas should fill predictive indicators and classifications under the ROC curve. The Youden index was used to define the ROC cut-off values. The significance of each analysis was increased by 95%. Data were applied through MedCalc Statistical Software version 16.4.3 (MedCalc Software bvba, Ostend, Belgium). A chi-square test was used for the statistical analysis of distribution differences of categorical data between groups. All statistical analyses were performed with PASW® v 18.0 for Windows®. The results had statistical significance at p < 0.05.

### Informed consent

All patients signed informed consent.

## Results

### Clinical and demographic characteristics

The case group comprised 75 patients (60 men and 15 women) with a mean age of 63.15 ± 11.64 years. Seventy (55.10%) patients of the case group received less than 2 Brazilian minimum wages (250 dollars) per month. Most patients (N = 65, 88%) did not complete elementary school (9 years of schooling). Regarding RT doses, 68% (N = 51) of the patients from the control group received more than 64 Gy during the RT. In the case group 58 (77.3%) patients presented OM grade 2, 8 (10.7%) presented grade 3 and 3 (2.7%) presented grade 4 (Supplementary Material 3). Taken all patients together 68 (90.6%) OM at some point during treatment with HNSCC.

**Figure 1:**
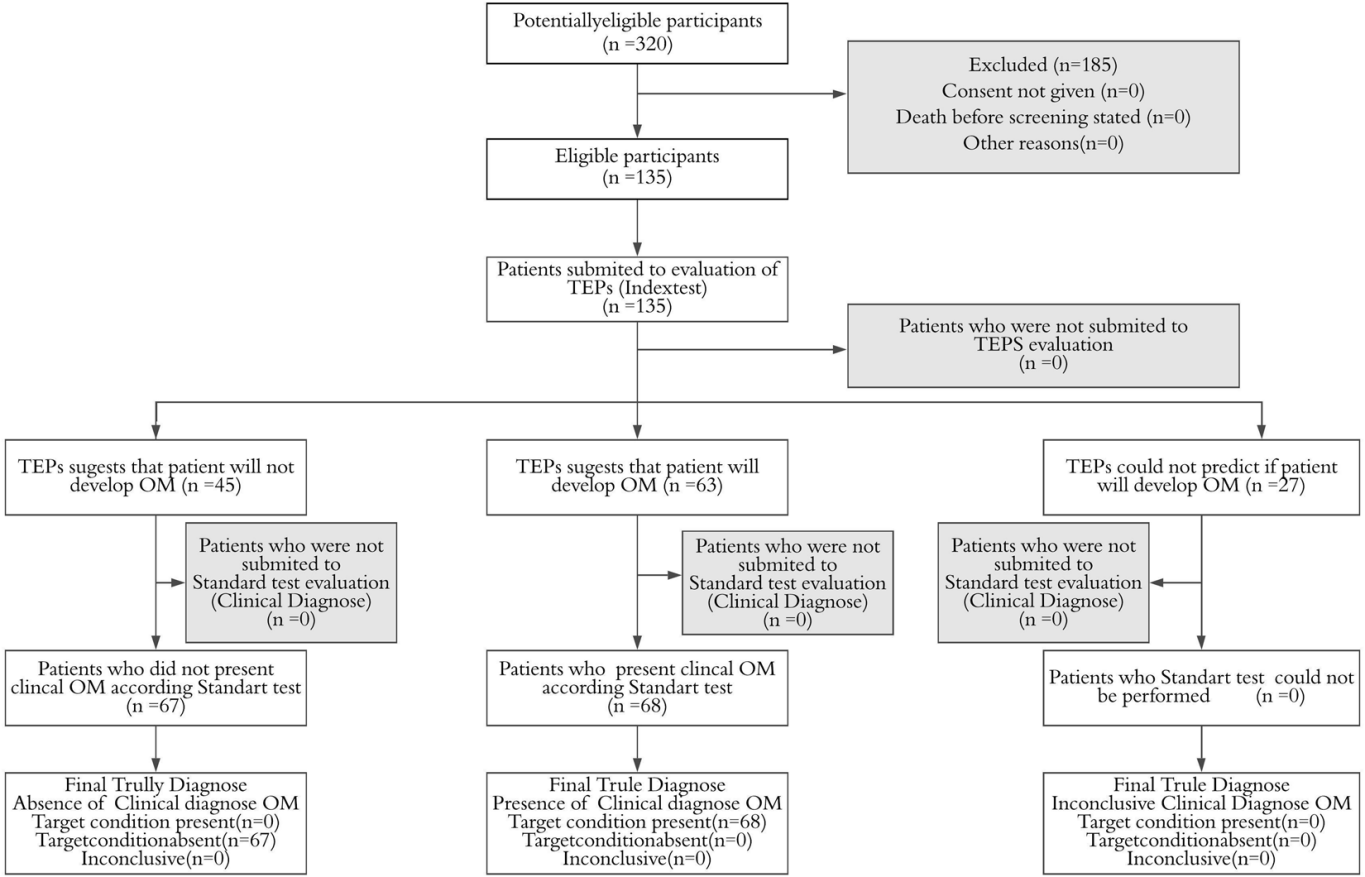
Flow diagram. Initially, 320 patients were potentially eligible for the study. Nevertheless, 185 did not meet the inclusion criteria. The study was performed with 135 patients. All 135 were submitted to the local and systemic TEPs evaluation, which was an index test. The standard test was the clinical diagnose of Oral Mucositis. All 135 patients were submitted to both index tests and standardized tests. The final diagnoses were the conclusion if the patient develops or not OM. According to TEPs, 48 patients will not develop mucositis. However, 67 patients did not develop OM. TEPs suggested that 63 patients will develop OM, while 68 indeed developed OM. TAPs field to conclude 27 patients.

The control group comprised 60 patients (50 men and 10 women), slightly older than the case group, with a mean age of 69.32 ± 12.94 years. The family income was lower than 2 Brazilian minimum wages (250 dollars) per month for 57 (95%) patients of the control group. Only six patients (10%) completed elementary school. Similar to the case group, 41 (68.33%) patients of the control group received more than 64 Gy. The groups did not differ in demographic and clinical characteristics (Table 2). The flow diagram of the study is presented in Fig. 1.

**Table 2:** Clinical characteristics of the case and control groups.

	Case		Control		Total	*p-v*alue
	N	%	N	%	N	
***Radiation Therapy Dose***						
≤64 Gy	24	55.8%	19	44.2%	43	
> 64 Gy	51	55.4%	41	44.6%	92	0.97
***Distant Metastasis (M)***						
M0	18	51.4%	17	48.6%	35	
M1	1	50.0%	1	50.0%	2	
MX	56	57.1%	42	42.9%	98	0.83
***Sex***						
Male	60	54.5%	50	45.5%	110	
Female	15	60.0%	10	40.0%	25	0.62
***Age (years)***						
Range	38–90		36–94			
Mean (SD)	63.15	(11.64)	69.32	(12.94)		0.43
***Family Income***						
<2 minimum wages	70	55.1%	57	44.9%	127	
>2 minimum wages	5	62.5%	3	37.5%	8	0.68
***Schooling***						
None	18	43.9%	23	56.1%	41	
Incomp. Elementary school	48	63.2%	28	36.8%	76	
Complete Elementary school	4	36.4%	4	63.6%	11	
Complete High school/Incomplete Higher Education	5	71.4%	2	28.6%	7	0.15

Abbreviations: RT, radiation therapy. 2 Brazilian minimum wages (250 dollars). Elementary school (9 years of schooling).

### Radiation therapy changes local tissue resistance

**Figure 2:**
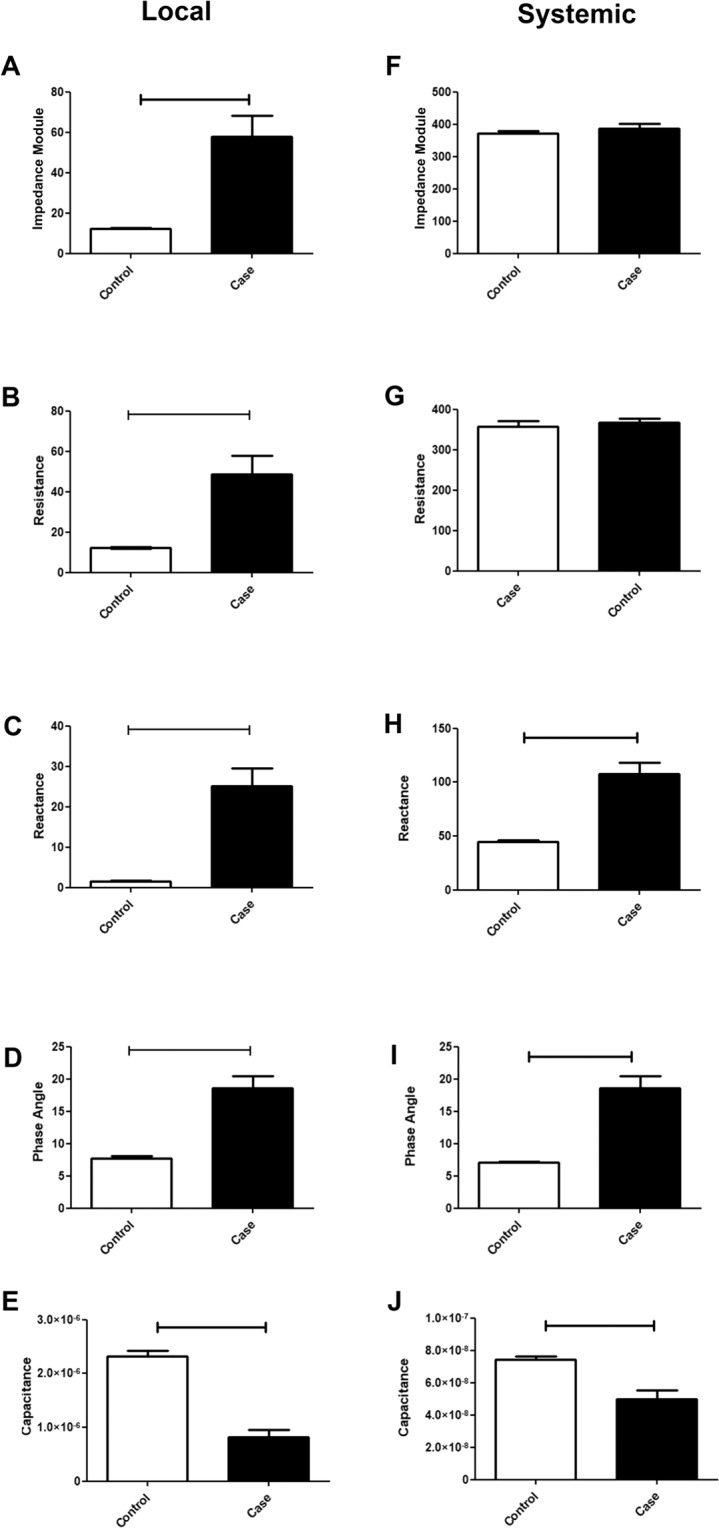
Systemic and local electrical parameters in case and control groups. The black bars represent the case group, while the white bars represent the control group. The bar charts depict the means; the error bars represent the standard deviation. The horizontal bars indicate significant *p* values.

In the current study, only measurements of TEPs after one week of RT were used to compare with the control group. It was observed that the case group after one week of RT presented differences in all local TEPs in comparison to the control group (Fig. 2A–E). The case group presented increasing in the local impedance module (Fig. 2A), resistance (Fig. 2B), reactance (Fig. 2C), and phase angle (Fig. 2D), after one week of RT in comparison to control group. The case group also presented an increase in systemic reactance and phase angle (Fig. 2H,I respectively) in comparison to the control group. Interestingly enough, the case group presented a reduction of both local (Fig. 2E) and systemic capacitance (Fig. 2J) in comparison to control. However, no differences between case and control groups were detected in the systemic impedance module and resistance (Fig. 2F,G).

### Local tissue resistance parameters indicate oral mucositis grade

**Figure 3:**
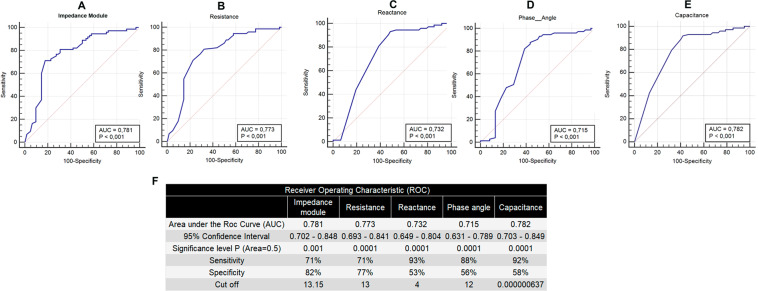
Receiver Operating Characteristic (ROC) curve of electrical parameters. Gold Standard by the curve for impedance module, resistance, reactance, phase angle, and capacitance (**A–E**, respectively).

The local electrical resistance parameters were changed by RT. Moreover, the evaluation of local TEPs in the first week of RT was able to predict the development of OM grade (Table 3). The first weekly measurements of local conductive parameters were used to perform ROC analysis to define the ability of electrical parameters to predict OM and determine the cut-off of impedance module (13.15), resistance (13.00), reactance (4.00), phase angle (12.00), and capacitance (6.37 × 10^−7^) to predict OM (Fig. 3A,B). Despite similar areas under the ROC curve, local impedance module and resistance present a better set of specificity and sensitivity than the other parameters.

**Table 3:** Correlation between tissue electrical parameters and oral mucositis.

TEP	Clinical Diagnose	Cutoff		Pearson test	*p*-value
*Impedance module*	*Oral mucositis*	≤*13.15*	>*13.15*		
	Yes	9	53		
	No	44	29	29.437	**0.0001**
*Resistance*	*Oral mucositis*	≤*13*	>*13*		
	Yes	14	48		
	No	52	21	31.759	**0.0001**
*Reactance*	*Oral mucositis*	≤*4*	>*4*		
	Yes	30	32		
	No	68	5	33.765	**0.0001**
*Phase angle*	*Oral mucositis*	≤*12*	>*12*		
	Yes	27	35		
	No	63	10	25.576	**0.0001**
*Capacitance*	*Oral mucositis*	≤*6.37E-07*	>*6.37E-07*		
	Yes	36	26		
	No	6	67	49.097	**0.0001**
*Impedance module*	*Oral mucositis grade*	≤*13.15*	>*13.15*		
	0	44	23		
	2	8	49		
	3	1	9		
	4	0	1	39.045	**0.0001**
*Resistance*	*Oral mucositis grade*	≤*13*	>*13*		
	0	52	15		
	2	13	44		
	3	1	9		
	4	0	1	44.648	**0.0001**
*Reactance*	*Oral mucositis grade*	≤*4*	>*4*		
	0	65	2		
	2	27	30		
	3	6	4		
	4	0	1	41.760	**0.0001**
*Phase angle*	*Oral mucositis grade*	≤*12*	>*12*		
	0	61	6		
	2	24	33		
	3	5	5		
	4	0	1	36.642	**0.0001**
*Capacitance*	*Oral mucositis grade*	≤*6.37E-07*	>*6.37E-07*	..	
	0	37	30		
	2	5	52		
	3	0	10		
	4	0	1	50.971	**0.0001**

Significant *p* values are in bold. Tissue Electrical Parameter (TEPs). All TEPs (Impedance module, Resistance, Reactance, Phase angle, Capacitance) were associated with the development of oral mucositis (OM) and the worse grade of OM.

## Discussion

Head and neck squamous cell carcinoma, an aggressive neoplasm, poses a significant public health issue^33^, with developing countries showing increased incidence rates in the last year^29^. Treatment modalities include surgery, RT, chemotherapy^1^, or combination therapy, depending on the TNM classification^1,2^. Treatment of HNSCC is associated with sequelae such as OM, radiodermatitis, and xerostomia^11,12,34^. The incidence of severe OM is responsible for RT interruptions^12^, that may affect RT fraction regimen^13^, resulting in decreased local control rates and reduced survival^14,15^. Strategies to prevent oral mucositides include cryotherapy^35^, low-level laser therapy^36^, and two-phase intensity-modulated radiation therapy^37^. Currently, there is no diagnostic tool to predict OM in cancer patients^12^.

Similarly, there are no diagnostic tools to predict the RT side effects^1^ Thus, we aimed to use initial tissue electrical parameters to estimate OM in HNSCC patients receiving RT. Tissue electrical parameters are widely used to evaluate body composition, lymphedema, and obesity^20,24,38^. Most of the studies on tissue electrical parameters in HNSCC were performed to study body composition and used only bioimpedance^18,22^. Recently, a study examined an association between the status of hydration and systemic impedance with OM^26^. However, other tissue electrical parameters such as resistance, reactance, phase angle, and capacitance have not yet been evaluated in the literature^18,22,26^. In the current study, the local measure of impedance module resistance, reactance, phase angle, and capacitance changed during RT.

The ROC curve demonstrated that all local tissue electric parameters were useful to differentiate cases from control. Furthermore, the tissue electrical parameters predicted post HNSCC treatment OM. Moreover, the values of the tissue electrical parameters measured in the first week of RT predicted the appearance of OM. This suggested that local tissue electrical parameters could be useful in stratifying patients with an increased risk for OM and determining preventive strategies to avoid poor prognosis. It is essential to highlight that data of the current study suggest that the tissue electrical parameters of the first week could be used to predict future side effects of RT. Currently, there is no available tool to predict OM during cancer therapy. The current study is the first trial to demonstrate that local tissue electrical parameters can predict oral mucositis in HNSCC. Prediction of OM will identify patients needing preventive interventions or treatment.

HNSCCs are known to contain abundant hypoxic areas^39,41–44^. However, studies have demonstrated that RT reduces the *in vivo* and *in vitro* levels of HIF-1α, miR-210, and LDH^40–44^. It is well-known that RT promotes ischemia-reperfusion injury^25^. In the current study, local impedance module, resistance, reactance, and phase angle were higher in irradiated anatomical sites. In contrast, local capacitance reduced with RT. The divergence in capacitance was expected because, by definition, impedance is directly proportional to frequency, while capacitance is inversely proportional to frequency. In corroboration with the current study, it was observed that there was an increase in the impedance parameters under ischemia and ischemia-reperfusion conditions^19^. Currently, there is no clinically approved tool to predict the success of RT^1^. In the future, the electrical parameters may also be useful to predict the effects of RT.

The main limitation of the current study was that it was conducted at a single center. Single-center studies usually present small and homogeneous patient populations. At the same time, the current study used two groups with similar clinical and demographic characteristics.

In conclusion, local tissue electrical parameters measured at the first RT week can be useful tools to predict oral mucositis.

## Supplementary information

Supplementary material.
