# Supplementary material for: Local tissue electrical parameters predict oral mucositis in HNSCC patients: A diagnostic accuracy double-blind, randomized controlled trial
Source: Sci Rep. 2020 Jun 12;10:9530. doi: 10.1038/s41598-020-66351-9 (PMC7293345; doi:10.1038/s41598-020-66351-9)
Supplement: Supplementary file 1 — Supplementary material. [file 41598_2020_66351_MOESM1_ESM.pdf]

Local tissue electrical parameters predict oral mucositis in HNSCC patients: A diagnostic accuracy double-blind randomized controlled trial

Gabriela Luize Guimarães Sanches[1], Agna Soares da Silva Menezes[1], Laércio Ives Santos[4],Cristina Paixão Durães[1], Larissa Lopes Fonseca[1], Marcelo Perim Baldo[3], Thais de Oliveira Faria[3], Luciano Alves de Araújo Andrade[6], Petr Iakovlevitch Ekel[7], Sérgio Henrique Sousa Santos[4], Alfredo Maurício Batista de Paula[1], Lucyana Conceição Farias[1], Marcos Flávio Silveira Vasconcelos D'Angelo[2] and André Luiz Sena Guimarães[1,3, 6#]

[1]Department of Dentistry,[2]Department of Computer Science and Department of Pathophysiology[3], Universidade Estadual de Montes Claros, Montes Claros, Minas Gerais, Brazil.
[4]Instituto Federal do Norte de Minas Gerais, Montes Claros, Minas Gerais, Brazil.
[5]Institute of Agricultural Sciences, Universidade Federal de Minas Gerais (UFMG), Montes Claros, Minas Gerais, Brazil.
[6]Dilson Godinho Hospital, Montes Claros, Minas Gerais, Brazil.
[7]Pontifícia Universidade Católica de Minas Gerais, Programa de Pós-Graduação em Engenharia Elétrica, Belo Horizonte, Minas Gerais, Brazil.

Corresponding author:
André Luiz Sena Guimarães
Universidade Estadual de Montes Claros. Hospital Universitário Clemente Faria. Laboratório de Pesquisa em Saúde, 562 Av. Cula Mangabeira Santo Expedito. Montes Claros, MG. Brazil Zip code: 39401-001
E-mail: andreluizguimaraes@gmail.com

Supplementary material 1:
STARD checklists

Section & Topic	No	Item	Reported on page #
**TITLE OR ABSTRACT**			
	**1**	Identification as a study of diagnostic accuracy using at least one measure of accuracy<br>(such as sensitivity, specificity, predictive values, or AUC)	1
**ABSTRACT**			
	**2**	Structured summary of study design, methods, results, and conclusions<br>(for specific guidance, see STARD for Abstracts)	2
**INTRODUCTION**			
	**3**	Scientific and clinical background, including the intended use and clinical role of the index test	3
	**4**	Study objectives and hypotheses	3
**METHODS**			
Study design	**5**	Whether data collection was planned before the index test and reference standard<br>were performed (prospective study) or after (retrospective study)	5
Participants	**6**	Eligibility criteria	5-6
	**7**	On what basis potentially eligible participants were identified<br>(such as symptoms, results from previous tests, inclusion in registry)	5-6
	**8**	Where and when potentially eligible participants were identified (setting, location and dates)	5
	**9**	Whether participants formed a consecutive, random or convenience series	5
Test methods	**10a**	Index test, in sufficient detail to allow replication	7
	**10b**	Reference standard, in sufficient detail to allow replication	6-7
	**11**	Rationale for choosing the reference standard (if alternatives exist)	
	**12a**	Definition of and rationale for test positivity cut-offs or result categories of the index test, distinguishing pre-specified from exploratory	-
	**12b**	Definition of and rationale for test positivity cut-offs or result categories of the reference standard, distinguishing pre-specified from exploratory	7
	**13a**	Whether clinical information and reference standard results were available<br>to the performers/readers of the index test	5
	**13b**	Whether clinical information and index test results were available<br>to the assessors of the reference standard	-
Analysis	**14**	Methods for estimating or comparing measures of diagnostic accuracy	7
	**15**	How indeterminate index test or reference standard results were handled	7
	**16**	How missing data on the index test and reference standard were handled	7
	**17**	Any analyses of variability in diagnostic accuracy, distinguishing pre-specified from exploratory	7
	**18**	Intended sample size and how it was determined	7
**RESULTS**			
Participants	**19**	Flow of participants, using a diagram	8
	**20**	Baseline demographic and clinical characteristics of participants	8
	**21a**	Distribution of severity of disease in those with the target condition	8
	**21b**	Distribution of alternative diagnoses in those without the target condition	-
	**22**	Time interval and any clinical interventions between index test and reference standard	8

Test results	**23**	Cross tabulation of the index test results (or their distribution) by the results of the reference standard	8
	**24**	Estimates of diagnostic accuracy and their precision (such as 95% confidence intervals)	8
	**25**	Any adverse events from performing the index test or the reference standard	8
**DISCUSSION**			
	**26**	Study limitations, including sources of potential bias, statistical uncertainty, and generalisability	9-10
	**27**	Implications for practice, including the intended use and clinical role of the index test	9-10
**OTHER INFORMATION**			
	**28**	Registration number and name of registry	5
	**29**	Where the full study protocol can be accessed	-
	**30**	Sources of funding and other support; role of funders	1

Supplementary material 2:
The schematic illustration of measurements.

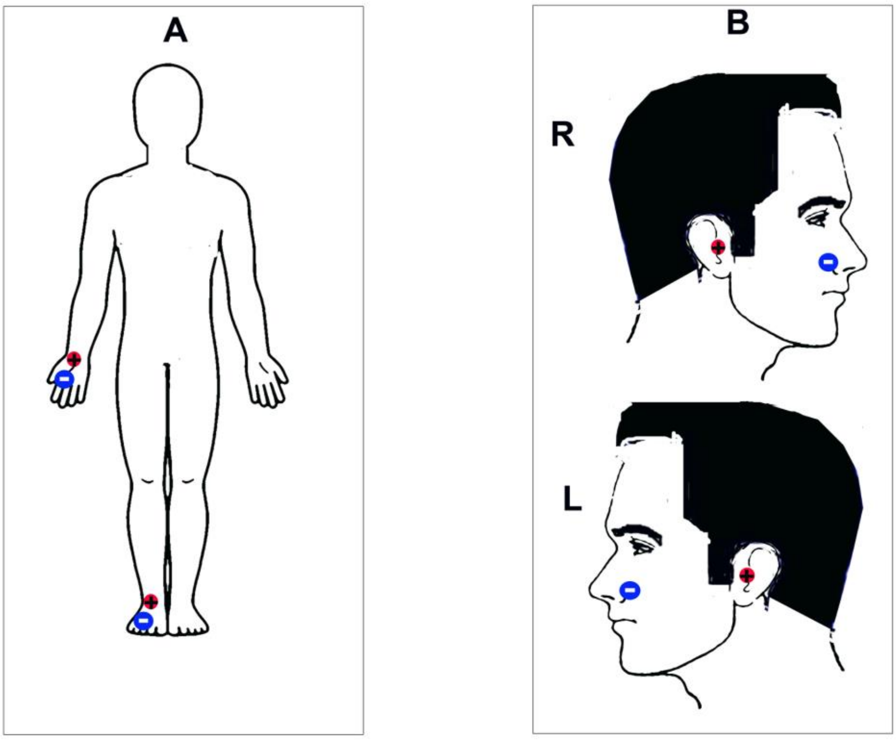

A represents systemic mesure and B the local measurements. L left and R right

Supplementary material 3:

Distribution of worse Oral Mucositis Grade between case and control groups during HNSCC treatment.

	Group			
	*Case		**Control*	
Worse Oral Mucositis Grade	N	%	N	%
0	7	9.3	60	100.0
1	0	0.0	0	0.0
2	58	77.3	0	0.0
3	8	10.7	0	0.0
4	2	2.7%	0	0.0

* Case group were evaluated weekly and treated when committed to Oral Mucositis. In the case of OM occurrence, treatment was initiated following standard protocols as described elsewhere (16,17). **Only one examination was performed in the control group.
